# Canonical vs. Grand Canonical Ensemble for Bosonic Gases under Harmonic Confinement

**DOI:** 10.3390/e26050367

**Published:** 2024-04-26

**Authors:** Andrea Crisanti, Luca Salasnich, Alessandro Sarracino, Marco Zannetti

**Affiliations:** 1 Dipartimento di Fisica, Università di Roma “La Sapienza”, Piazzale Moro 5, 00185 Roma, Italy; andrea.crisanti@uniroma1.it; 2 Dipartimento di Fisica e Astronomia “Galileo Galilei” and Padua QTech Center, Università di Padova, Via Marzolo 8, 35131 Padova, Italy; 3 Istituto Nazionale di Fisica Nucleare, Sezione di Padova, Via Marzolo 8, 35131 Padova, Italy; 4 Istituto Nazionale di Ottica del Consiglio Nazionale Delle Ricerche, Via Nello Carrara 2, 50127 Sesto Fiorentino, Italy; 5 Dipartimento di Ingegneria, Università della Campania “Luigi Vanvitelli”, Via Roma 29, 81031 Aversa, Italy; alessandro.sarracino@unicampania.it; 6 Dipartimento di Fisica “Eduardo Caianiello”, Università di Salerno, Via Giovanni Paolo II 132, 84084 Salerno, Italy; mrc.zannetti@gmail.com

**Keywords:** statistical ensemble, Bose–Einstein condensation, photons

## Abstract

We analyze the general relation between canonical and grand canonical ensembles in the thermodynamic limit. We begin our discussion by deriving, with an alternative approach, some standard results first obtained by Kac and coworkers in the late 1970s. Then, motivated by the Bose–Einstein condensation (BEC) of trapped gases with a fixed number of atoms, which is well described by the canonical ensemble and by the recent groundbreaking experimental realization of BEC with photons in a dye-filled optical microcavity under genuine grand canonical conditions, we apply our formalism to a system of non-interacting Bose particles confined in a two-dimensional harmonic trap. We discuss in detail the mathematical origin of the inequivalence of ensembles observed in the condensed phase, giving place to the so-called grand canonical catastrophe of density fluctuations. We also provide explicit analytical expressions for the internal energy and specific heat and compare them with available experimental data. For these quantities, we show the equivalence of ensembles in the thermodynamic limit.

## 1. Introduction

Bose–Einstein condensation (BEC) was first experimentally observed with ultracold and dilute atomic gases in 1995 by several groups [1,2,3]. Since then, the phenomenon has been reproduced in a huge variety of systems [4], such as trapped alkali gases [5,6], rotating systems [7], quantum magnets [8], and so on. For all these systems, the number of atoms is conserved and the statistical properties are well described within the canonical ensemble (CE). More recently, however, and quite surprisingly, BEC has also been realized with effectively massive photons in a dye-filled optical cavity [9,10,11,12,13,14,15], where it is possible to work in a regime with a number of photons that are conserved only on average. In this case, the proper ensemble to describe the system features is the grand canonical ensemble (GCE).

The relation between CE and GCE is a general problem of statistical mechanics and the two ensembles are usually equivalent in the thermodynamic limit. This is the case when correlations are short-ranged and, due to the central limit theorem, fluctuations in extensive quantities become negligible for a large system size. However, there exist noteworthy exceptions, where computations performed in the two ensembles lead to different results. This occurs, for instance, in long-range systems [16], where the issue of ensemble inequivalence is very well studied. In the framework of systems of free bosons, this topic has been considered theoretically in several studies [17,18,19,20,21]. The remarkable result is that, while, in the CE, density fluctuations vanish in the thermodynamic limit as generally expected, in the GCE, they are macroscopic and remain finite upon decreasing the temperature, leading to the so-called grand canonical catastrophe, with an explicit negative connotation. However, the experimental realization of BEC in genuine grand canonical conditions has confirmed the theoretical predictions obtained in the GCE, making the issue related to the inequivalence of ensembles in this context a heated topic of debate. In a previous paper [22], building on an analogy with the celebrated spherical model of a ferromagnet introduced by Berlin and Kac [23], some of us have interpreted the physical meaning of the grand canonical catastrophe predicted for a homogeneous ideal Bose gas as a phenomenon of the condensation of fluctuations [24].

In this paper, before considering the experimentally relevant case of quantum gases of non-interacting bosons confined in a two-dimensional harmonic trap, we first present an accurate discussion of the relation between canonical and grand canonical ensembles in the general framework of statistical mechanics. In particular, we bring to the fore the mathematical aspects related to the (in-)equivalence between ensembles, focusing on the behaviors arising in the thermodynamic limit. We re-derive some important results obtained by Ziff et al. [17] for ideal gases, following an alternative approach, based on the theory of singular perturbations and boundary layer problems [25]. We then apply our formalism to the case of a free gas of bosons confined by a parabolic potential and investigate several physical features. We find that, in the thermodynamic limit, in the Bose-condensed phase, the density fluctuations and the spatial density–density correlation function have a quite different behavior in the two ensembles. In contrast, other quantities, such as the critical temperature, the condensate fraction, internal energy, and the specific heat behave similarly. For the last two quantities, we provide explicit analytical expressions that very well compare to the experimental data reported in [12].

This paper is structured as follows: in Section 2, we develop the general formalism to connect CE to GCE with the kernel introduced in ref. [26]. In Section 3, we discuss the problem of Bose–Einstein condensation for an ideal gas of massive bosons confined in a two-dimensional harmonic potential. In Section 3, we compare the theoretical predictions of the CE and GCE for several quantities. We discuss in particular the issue related to the grand canonical catastrophe and its meaning. We also show that our theory reproduces the experimental results of internal energy and specific heat of the photons in an optical cavity quite well. Conclusions are drawn in Section 4. It is important to stress that, in our two-dimensional problem, there is true BEC due to the presence of the harmonic confinement, while there is no Berezinskii–Kosterlitz–Thouless phase transition because the bosons are not interacting.

## 2. Relation between Canonical and Grand Canonical Ensembles

We start by discussing some standard results of statistical mechanics. This allows us to introduce the general problem and to fix the notation that will be used throughout the paper. A general relation between the CE and GCE can be established at different levels. We start by considering the connection between the partition functions. We denote by $\sigma =(\sigma_{1},\ldots ,\sigma_{N})$ the state of a generic system, and by H(σ) its Hamiltonian. The natural variables of the CE are the temperature $k_{B}T=\beta^{-1}$, the volume *V*, and the number of elements *N*. It is important to underline that, in the subsequent sections, which investigate the bosonic system under harmonic confinement, the volume *V* will be an “effective volume” related to the frequency of the harmonic potential. The probability density of a configuration σ is

(1) $$P_{C}\left(\sigma |\beta ,V,N\right)=\frac{1}{Z_{C}\left(\beta ,V,N\right)}\;e^{-\beta H(\sigma )},$$

where

(2) $$Z_{C}\left(\beta ,V,N\right)=\sum_{\left\{\sigma \right\}}\;e^{-\beta H(\sigma )}$$

is the canonical partition function, where the sum extends over all possible system configurations.

In the GCE, the natural variables are the temperature $k_{B}T=\beta^{-1}$, the volume *V*, and the fugacity $z=e^{\beta \mu }$, where μ is the chemical potential. The probability that the system is in the configuration σ with *N* elements is, in the GCE,

(3) $$P_{\mathrm{GC}}\left(\sigma ,N|\beta ,V,z\right)=\frac{z^{N}e^{-\beta H_{N}\left(\sigma \right)}}{Z_{\mathrm{GC}}\left(\beta ,V,z\right)},$$

where

(4) $$\begin{array}{ccc} Z_{\mathrm{GC}}\left(\beta ,V,z\right) & = & \sum_{N\geq 0}\;\sum_{\left\{\sigma \right\}}\;z^{N}\;e^{-\beta H_{N}\left(\sigma \right)}\\ & = & \sum_{N\geq 0}\;z^{N}Z_{C}\left(\beta ,V,N\right) \end{array}$$

is the grand canonical partition function defined for $ℜz<z_{0}$, where $z_{0}$ is a model-dependent parameter ensuring the convergence of the sum in Equation (4). We have added the subscript *N* to the Hamiltonian to indicate that $H_{N}\left(\sigma \right)$ refers to a system of *N* elements. From the above relation, one immediately has that the GCE partition function can be obtained as a z−transform of the CE partition function. The Relation (4) can be inverted as

(5) $$Z_{C}\left(N\right)=\frac{1}{2\pi i}\oint_{\Gamma }dz\;z^{-1-N}\;Z_{\mathrm{GC}}\left(z\right),$$

where Γ is a generic curve in the complex plane encircling the origin z=0 (to simplify the notation, we have only indicated the relevant variables).

### 2.1. The Kernel K(N|z)

The connection between the GCE and CE can also be established at the level of probability density. Indeed, writing

(6) $$P_{\mathrm{GC}}\left(\sigma ,N|z\right)=\frac{z^{N}\;Z_{C}\left(N\right)}{Z_{\mathrm{GC}}\left(z\right)}\;\frac{e^{-\beta H(\sigma )}}{Z_{C}\left(N\right)},$$

and using (1), one obtains

(7) $$P_{\mathrm{GC}}\left(\sigma ,N|z\right)=P_{C}\left(\sigma |N\right)\;K\left(N|z\right),$$

where

(8) $$K\left(N|z\right)=\frac{z^{N}\;Z_{C}\left(N\right)}{Z_{\mathrm{GC}}\left(z\right)}$$

is the kernel relating the two distributions, and represents the probability that the system consists of *N* elements for fixed *z*. Its explicit form depends on the specific model. Celebrated examples are discussed in [26] for the spherical model and in [17] for the homogeneous ideal Bose gas. We will consider the trapped ideal Bose gas in the following sections.

Finally, from the Relation (7), a connection between averages readily follows. Indeed, we have

(9) $${\langle f\left(\sigma \right)\rangle }_{\mathrm{GC}}\left(z\right)=\sum_{N}{\langle f\left(\sigma \right)\rangle }_{C}\left(N\right)\;K\left(N|z\right),$$

where we have denoted by ${\langle \ldots \rangle }_{\mathrm{GC}}$ and ${\langle \ldots \rangle }_{C}$ averages in the GCE and CE, respectively, and f(σ) is a generic function of the system configuration. The above equation can also be interpreted as the generating function of ${\langle f\left(\sigma \right)\rangle }_{C}$, as first introduced in [17]; see their Equation (2.34). As detailed in Appendix A, this relation can be formally inverted, yielding

(10) $${\langle f\left(\sigma \right)\rangle }_{C}\left(N\right)=\frac{1}{2\pi i}\;\oint_{\Gamma }\frac{dz}{z}\frac{{\langle f\left(\sigma \right)\rangle }_{\mathrm{GC}}\left(z\right)}{K(N|z)}.$$

### 2.2. Thermodynamic Limit

The discussion presented in the previous section can be simplified in the large *N* limit, leading to some more explicit general results. The thermodynamic limit is obtained by taking

(11) N≫1,V≫1butN/V=ρ=constant.

Rewriting the GCE partition function (4) as

(12) $$Z_{\mathrm{GC}}\left(z,V\right)=\sum_{N}e^{N\ln z+\ln Z_{C}\left(N,V\right)},$$

since $\ln Z_{C}\left(N,V\right)=O\left(N\right)$ as N≫1, the sum is dominated in the thermodynamic limit (11) by the term with the largest value of the exponent:

(13) $$Z_{\mathrm{GC}}\left(z,V\right)∼z^{N^{*}\left(z\right)}\;Z_{C}\left(N^{*}\left(z\right),V\right),$$

where

(14) $$N^{*}\left(z\right)=\underset{N}{arg max}[N\ln z+\ln Z_{C}\left(N,V\right)].$$

To evaluate $N^{*}\left(z\right)$, we rewrite Equation (12) as

(15) $$Z_{\mathrm{GC}}\left(z,V\right)=V\;\sum_{N}\;\Delta \rho \;e^{V\left[\rho \ln z+\frac{1}{V}\ln Z_{C}\left(\rho ,V\right)\right]},$$

where Δρ=1/V≪1 as V≫1, and, in the thermodynamic limit, we have

(16) $$Z_{\mathrm{GC}}\left(z,V\right)∼V\int d\rho \;e^{V\phi (\rho )},\;\;V\gg 1$$

with

(17) $$\phi \left(\rho \right)=\rho \ln z+\frac{1}{V}\ln Z_{C}\left(\rho ,V\right).$$

Note that, since $\ln Z_{C}\left(\rho ,V\right)=O\left(V\right)$ in the large volume limit, the second term on the right-hand side becomes independent of *V*. We will drop O(1/V) corrections in the following. The integral can then be evaluated using the Laplace method, expanding ϕ(ρ) as

(18) $$\phi \left(\rho \right)=\phi \left(\rho^{*}\right)+\frac{1}{2}\phi^{\prime \prime }\left(\rho^{*}\right){\left(\rho -\rho^{*}\right)}^{2}+O{\left(\rho -\rho^{*}\right)}^{3},$$

where $\rho^{*}$ is the solution of the stationary point equation

(19) $$\phi^{\prime }\left(\rho^{*}\right)=\ln z+\frac{1}{V}\frac{\partial }{\partial \rho^{*}}\ln Z_{C}\left(\rho^{*},V\right)=0.$$

Then, introducing $\sigma_{\phi }^{-2}=-{\left(\partial /\partial \rho^{*}\right)}^{2}\ln Z_{C}\left(\rho^{*},V\right)>0$, we have

(20) $$Z_{\mathrm{GC}}\left(z,V\right)∼V\;\sqrt{2\pi \sigma_{\phi }^{2}}\;e^{V\left[\rho^{*}\ln z+\frac{1}{V}\ln Z_{C}\left(\rho^{*},V\right)\right]},V\gg 1.$$

Writing $N^{*}\left(z\right)=V\rho^{*}$, and taking only the leading term, we recover Equation (13), with $N^{*}\left(z\right)$ given by the solution of

(21) $$N^{*}\left(z\right):\;\frac{\partial }{\partial N}\ln Z_{C}\left(N,V\right)=-\ln z.$$

Equation (13) implies that

(22) $$\ln Z_{\mathrm{GC}}\left(z,V\right)=\ln Z_{C}\left(N,V\right)+N\ln z,$$

where $N=N^{*}\left(z\right)$ is obtained from Equation (21). Equations (21) and (22) show that, in the thermodynamic limit (11), the $\ln Z_{\mathrm{GC}}\left(z,V\right)$ is the Legendre transform of $\ln Z_{C}\left(N,V\right)$ with respect to *N*.

We now consider the kernel K(N|z). From the previous discussion, it follows that, in the thermodynamic limit, for fixed *z*,

(23) $$\begin{array}{ccc} K(N|z) & = & \frac{z^{N}\;Z_{C}\left(N,V\right)}{Z_{\mathrm{GC}}\left(z,V\right)}=\frac{e^{V\phi (\rho )}}{V\int d\rho \;e^{V\phi (\rho )}}\\ & ∼ & \frac{e^{-\frac{1}{2\sigma_{\phi }^{2}}{\left(\rho -\rho^{*}\right)}^{2}}}{V\sqrt{2\pi \sigma_{\phi }^{2}}}, \end{array}$$

where, in the second equality, we have used Equation (17), and hence, since $\sigma_{\phi }^{2}=O\left(1/N\right)$,

(24) $$K\left(N|z\right)∼\Delta \rho \;\delta \left(\rho -\rho^{*}\right)∼\delta^{\mathrm{Kr}}\left(N-N^{*}\left(z\right)\right),$$

with $N^{*}\left(z\right)$ the solution of (21) and $\delta^{Kr}$ the Kronecker delta. This result is also discussed, for instance, in [17]—see their Equation (2.63)—and shows the equivalence of the ensembles in the thermodynamic limit. The same result can be obtained starting from the expression of $Z_{C}\left(N\right)$ as a transform of $Z_{\mathrm{GC}}\left(z\right)$, as reported in Appendix B of this paper. The above discussion shows that, for a fixed *z*, with $ℜz<z_{0}$, there exists a corresponding $N^{*}\left(z\right)$ and therefore there exists an equivalent canonical ensemble with $N=N^{*}\left(z\right)$.

The general findings obtained so far break down in specific cases, where the application of the Laplace method requires particular care. This can lead to different behaviors in different ensembles, as described in the next section.

## 3. Ideal Bose Gas in a Harmonic Trap

As a specific system, we consider a two-dimensional (2D) harmonically trapped system of non-interacting massive bosons. This model has been shown to provide a very good description of a photon gas confined in a high-finesse dye-filled microcavity, studied in the experiments [9,10,11,12,13,14,15], where Bose–Einstein condensation has been observed. In particular, the microcavity was realized by two curved mirrors, where photons were continuously absorbed and re-emitted by the dye medium, which is crucial for the equilibration. Due to a cavity length of the same order as the photon wavelength, a frequency gap between the longitudinal resonator modes was realized. Thus, the system consists of photons with a fixed longitudinal mode number that are allowed to populate several transversally excited cavity modes, making the photon gas effectively two-dimensional. The energy–momentum relation is described by a quadratic term, with an effective mass, and a trapping parabolic potential induced by the mirror curvature [10].

It has been shown that this experimental setup realizes bona fide grand canonical conditions where theoretical predictions can be tested [13,14,15]. We are interested in the different behaviors that can arise in different ensembles, in particular for what concerns the fluctuations in the occupation number of the condensed phase. In the following, we will apply the formalism introduced in the previous section to the system of trapped bosons in 2D. Our theoretical treatment differs from that presented in [9], where the photon number statistics are derived from a rate equation model in the stationary regime. Our analysis follows rather the analytical theory of Ziff et al. [17], developed for the case of a free ideal gas. The main difference relies on the derivation of the Kac kernel in the region below the critical temperature, which we present here for the case of a trapped Bose gas, following an alternative approach based on the theory of singular perturbations.

### 3.1. The Model

In second quantization, the Hamiltonian of the system can be written as [27]

(25) $$\hat{H}=\int d^{2}r\;{\hat{\psi }}^{†}\left(r\right)\left[-\frac{\hbar^{2}}{2m_{\mathrm{eff}}}\nabla^{2}+U\left(r\right)\right]\hat{\psi }\left(r\right)\;,$$

where $\hat{\psi }\left(r\right)$ (${\hat{\psi }}^{†}\left(r\right)$) is the bosonic field operator, which destroys (creates) a boson at the position r=(x,y), $m_{\mathrm{eff}}$ is the effective mass of each photon, which depends on the mirror separation and on the linear index of the refraction of the medium (see [10] for details), and

(26) $$U\left(r\right)=\frac{1}{2}m_{\mathrm{eff}}\omega^{2}\left(x^{2}+y^{2}\right)$$

is the 2D harmonic potential with trapping frequency ω. The local number density operator is given by

(27) $$\hat{\rho }\left(r\right)={\hat{\psi }}^{†}\left(r\right)\hat{\psi }\left(r\right),$$

while

(28) $$\hat{N}=\int d^{2}r\;\hat{\rho }\left(r\right)$$

is the total number operator.

The single-particle quantum mechanics in the 2D harmonic potential (26) are described by the stationary Schrödinger equation

(29) $$\left[-\frac{\hbar^{2}}{2m_{\mathrm{eff}}}\nabla^{2}+U\left(r\right)\right]\phi_{m}\left(r\right)=\epsilon_{m}\;\phi_{m}\left(r\right)\;,$$

where $m=(m_{x},m_{y})$ and the eigenvalues are given by $\epsilon_{m}=\hbar \omega \left(m_{x}+m_{y}+1\right)$ with $m_{x},m_{y}=0,1,2,\ldots$ being the two natural quantum numbers, while $\phi_{m}\left(r\right)$ are the corresponding orthonormal eigenfunctions. In particular, for the single-particle ground state, we have $\epsilon_{0}=\hbar \omega$ and

(30) $$\phi_{0}\left(r\right)=\frac{1}{\sqrt{\pi }l_{H}}e^{-\frac{x^{2}+y^{2}}{2l_{H}^{2}}},$$

with $l_{H}=\sqrt{\frac{\hbar }{m_{\mathrm{eff}}\omega }}$ the characteristic length of the harmonic confinement.

The field operator $\hat{\psi }\left(r\right)$ can be expanded in any orthonormal basis. Here, we choose the basis of the eigenfunctions $\phi_{m}\left(r\right)$ of the single-particle problem of Equation (29), i.e.,

(31) $$\begin{array}{ccc} \hat{\psi }\left(r\right) & = & \sum_{m\in N^{2}}{\hat{a}}_{m}\phi_{m}\left(r\right), \end{array}$$

(32) $$\begin{array}{ccc} {\hat{\psi }}^{†}\left(r\right) & = & \sum_{m\in N^{2}}{\hat{a}}_{m}^{†}\phi_{m}^{*}\left(r\right), \end{array}$$

where ${\hat{a}}_{m}$ and ${\hat{a}}_{m}^{†}$ are the ladder operators which, respectively, destroy and create a boson in the single-particle quantum state $\phi_{m}\left(r\right)$. Inserting the Formulas (31) and (32) into Equation (25), and using Equation (29) and the orthonormal property, we obtain

(33) $$\hat{H}=\sum_{m\in N^{2}}\epsilon_{m}\;{\hat{N}}_{m}\;,$$

where

(34) $${\hat{N}}_{m}={\hat{a}}_{m}^{†}{\hat{a}}_{m}$$

is the number operator of the single-particle quantum state. Similarly, we find

(35) $$\hat{\rho }\left(r\right)=\sum_{m,m^{\prime }\in N^{2}}{\hat{a}}_{m}^{†}{\hat{a}}_{m^{\prime }}\phi_{m}^{*}\left(r\right)\phi_{m^{\prime }}\left(r\right),$$

and clearly

(36) $$\hat{N}=\sum_{m\in N^{2}}{\hat{N}}_{m}\;$$

is the total number operator of the bosons under investigation.

The Fock state $|n_{m}\rangle$ represents the occupation number quantum state describing the number *n* of bosons that are in the single-particle quantum state |m〉. It satisfies the eigenvalue equation

(37) $${\hat{N}}_{m}|n_{m}\rangle =n_{m}|n_{m}\rangle \;.$$

We can also introduce a generic multi-mode Fock state [22,27] of our problem associated with the set of occupation numbers

(38) $$n=\left\{n_{m}\right\}=\left(n_{00},n_{01},n_{10},n_{11},\;\ldots \right),$$

as

(39) $$|n\rangle =\prod_{m\in N^{2}}|n_{m}\rangle =|n_{00}\rangle \;|n_{01}\rangle \;|n_{10}\rangle \;|n_{11}\rangle \;\ldots$$

This state is characterized by $n_{00}$ photons in the single-particle state |00〉, $n_{01}$ photons in the single-particle state |01〉, $n_{11}$ photons in the single-particle state |11〉, et cetera. These multi-mode Fock states can be used to obtain the following spectral resolution of the identity

(40) $$\hat{1}=\sum_{n}|n\rangle \langle n|\;.$$

### 3.2. Grand Canonical Formulation

In order to describe the phenomenon of the Bose–Einstein condensation in this system, we start from the GCE, where the density operator is

(41) $$\hat{D}=e^{-\beta (\hat{H}-\mu \hat{N})},$$

and the probability of the set of occupation numbers n is given by

(42) $$P_{\mathrm{GC}}\left(n\right)=\frac{1}{Z_{\mathrm{GC}}}\langle n|\hat{D}|n\rangle =\frac{1}{Z_{\mathrm{GC}}}e^{-\beta \sum_{m}\left(\epsilon_{m}-\mu \right)n_{m}},$$

where

(43) $$Z_{\mathrm{GC}}=Tr\left[e^{-\beta (\hat{H}-\mu \hat{N})}\right]=\prod_{m}{\left[1-e^{-(\beta \lambda_{m}+\kappa )}\right]}^{-1}$$

and

(44) $$\begin{array}{c} \lambda_{m}=\epsilon_{m}-\epsilon_{0}, \end{array}$$

(45) $$\begin{array}{ccc} & & \kappa =\beta (\epsilon_{0}-\mu ). \end{array}$$

#### 3.2.1. Equation of State

The grand canonical thermal average of the total number operator reads

(46) $${\langle \hat{N}\rangle }_{GC}=\frac{Tr[\hat{N}e^{-\beta (\hat{H}-\mu \hat{N})}]}{Z_{GC}}=-\frac{\partial }{\partial \kappa }\ln Z_{\mathrm{GC}},$$

where

(47) $$-\ln Z_{\mathrm{GC}}=\sum_{m}\;\ln\left[1-e^{-(\beta \lambda_{m}+\kappa )}\right],$$

and

(48) $$\langle {\hat{N}}_{m}\rangle =\frac{1}{e^{\left(\beta \lambda_{m}+\kappa \right)}-1}.$$

In order to proceed further, we now introduce the definition of the thermodynamic limit for our system of bosons confined by a harmonic potential. Following [6], we consider the conditions

(49) $$N\gg 1,\;\omega \ll 1,\;N\hbar^{2}\omega^{2}=\rho =\mathrm{finite},$$

where we have included *ℏ* into the definition of the pseudo-density ρ for simplicity. To keep the notation formally similar to the more familiar case of a system of particles in a box of volume *V*, as considered in the previous sections, we now introduce a pseudo-volume $V=1/{\left(\hbar \omega \right)}^{2}$ so that the condition of the thermodynamic limit can be also written as N≫1 and V≫1, with ρ=N/V fixed.

Next, separating from the sum in Equation (47) the m=0 term, we have

(50) $$\ln Z_{\mathrm{GC}}\left(\beta ,s\right)=-\ln\left(1-s\right)-\frac{V}{\beta^{2}}J\left(s\right),$$

where we have defined $s=e^{-\kappa }=ze^{-\beta \epsilon_{0}}\leq 1$, which plays the role of a fugacity in a rescaled reference frame with zero lowest energy. In the following, we will find it more convenient to consider the thermodynamic quantities as a function of *s* rather than *z*. The function $J\left(s\right)\equiv -g_{3}\left(s\right)$, where $g_{n}\left(s\right)$ denotes the Bose functions [17], represents the sum over the excited states and is given by

(51) $$J\left(s\right)=\int_{0}^{+\infty }dy\;y\;\ln\left(1-s\;e^{-y}\right).$$

Using (50), we have

(52) $${\langle \hat{N}\rangle }_{\mathrm{GC}}=s\frac{\partial }{\partial s}\ln Z_{\mathrm{GC}}\left(\beta ,s\right)=\frac{s}{1-s}+N_{1}\left(\beta ,s\right),$$

where

(53) $$N_{1}\left(\beta ,s\right)=\frac{V}{\beta^{2}}I\left(s\right),$$

with

(54) $$I\left(s\right)\equiv g_{2}\left(s\right)=-s\frac{\partial }{\partial s}J\left(s\right)=\int_{0}^{+\infty }dy\;\frac{y}{s^{-1}\;e^{y}-1}.$$

#### 3.2.2. Bose-Einstein Phase Transition

The standard argument leading to the phenomenon of condensation is as follows: since $I^{\prime }\left(s\right)>0$, the function I(s) is a monotonous increasing function of *s*, so that, for any β, one has

(55) $$N_{1}\left(\beta ,s\right)\leq N_{c}\left(\beta \right)=\frac{V}{\beta^{2}}I\left(1\right).$$

If I(1) is finite, as in our case $I\left(1\right)=\pi^{2}/6$, $N_{c}\left(\beta \right)$ is a finite decreasing function of β so that, for any fixed integer number *N*, there exists a finite $\beta_{c}\left(N\right)$, the inverse critical temperature, such that

(56) $$\beta_{c}\left(N\right):\;N_{c}\left(\beta_{c}\right)=N.$$

To address the issue of the ensemble (in)equivalence in the condensed phase, we consider the condition

(57) $${\langle \hat{N}\rangle }_{\mathrm{GC}}=N,$$

where *N* now plays the role of a control parameter in the corresponding CE (see also the derivation reported in Appendix B leading to Equation (A18)). Then, for $N>N_{c}\left(\beta \right)$, or $\beta >\beta_{c}\left(N\right)$, the contribution from the lowest energy level in Equation (52) cannot be neglected. In terms of the pseudo-density ρ, the conditions (55) and (56) become

(58) $$\rho_{1}\left(\beta ,s\right)\leq \rho_{c}\left(\beta \right)=\frac{1}{\beta^{2}}I\left(1\right),$$

and

(59) $$\beta_{c}\left(\rho \right):\;\rho_{c}\left(\beta_{c}\right)=\rho .$$

Then, from the condition (57), one has

(60) $$\rho =\frac{1}{V}\frac{s}{1-s}+\rho_{1}\left(\beta ,s\right),$$

where $\rho_{1}\left(\beta ,s\right)=N_{1}\left(\beta ,V,s\right)/V$. We stress that, in Equation (59), the density ρ is an arbitrary parameter, as *N* in Equation (56). In Equation (60), the density ρ on the left-hand side refers to the canonical ensemble (β,V,N) while the quantities on the right-hand side refer to the grand canonical ensemble (β,V,s).

From this structure, it is clear that, in order to study the thermodynamic limit correctly, we must distinguish two regions: the first, where 1−s=O(1) as V≫1, which is appropriate for $\beta <\beta_{c}$, and the second, where 1−s=O(1/V) as V≫1, describing the case $\beta >\beta_{c}$. In singular perturbation theory, this case represents an instance of a *boundary layer problem* with a *boundary layer* at s=1. The regions are called, respectively, the *outer* and *inner* region in boundary layer theory. We note that the results discussed in Section 2.2 hold for $\beta <\beta_{c}$ above the critical temperature.

### 3.3. The Kac Kernel

We now study the behavior of the Kac kernel, which represents the mathematical transformation connecting corresponding quantities in the two ensembles. This allows us to address the issue related to the ensemble equivalence, in the case $\beta >\beta_{c}$. The explicit form of the kernel was first derived in [17] in the case of a uniform system. Here, we obtain the same results for the case of the trapped gas following an alternative approach. In particular, we have to consider what happens in the region where 1−s=O(1/V) as V≫1, and this can be achieved by performing a convenient change in variable prior to the thermodynamic limit. To be specific, let us rewrite K(N|z) in the following form (see Equation (A6) in the Appendix A for details):

(61) $$K\left(N^{\prime }|z\right)=\frac{1}{2\pi i}\oint_{\Gamma }d\xi \;\xi^{-1-N^{\prime }}\;\frac{Z_{\mathrm{GC}}\left(\beta ,\xi z\right)}{Z_{\mathrm{GC}}\left(\beta ,z\right)}.$$

We use $N^{\prime }$ to emphasize that it is a running argument unrelated to *z*. The fugacity *z* is related to ${\langle \hat{N}\rangle }_{GC}$ imposing the condition (60) but substituting ρ with $\bar{\rho }={\langle \hat{N}\rangle }_{GC}/V$, namely

(62) $$\bar{\rho }=\frac{1}{V}\frac{s}{1-s}+\rho_{1}\left(\beta ,s\right).$$

We introduce the variable η through the relation $s=ze^{-\beta \epsilon_{0}}=1-\eta /V$, where η=O(1) as V≫1. A similar change in variable must be carried out for ξ. We have seen in Section 2.2 that, in the limit of large *N*, the integral is dominated by a path parallel to the imaginary axis. This remains true also for $\beta >\beta_{c}$ because ${\left(\partial /\partial \xi \right)}^{2}\ln Z_{\mathrm{GC}}\left(\beta ,\xi z\right)>0$. Thus, we write ξ=1+iv/V, where v=O(1) as V≫1. Using (50) and

(63) $$\xi s=\left(1+iv/V\right)\left(1-\eta /V\right)∼1-\left(\eta -iv\right)/V+O\left(1/V^{2}\right),$$

we have

(64) $$\begin{array}{cc} \ln\frac{Z_{\mathrm{GC}}\left(\beta ,\xi z\right)}{Z_{\mathrm{GC}}\left(\beta ,z\right)} & ∼-\ln\left((\eta -iv)/V\right)+\ln\left(\eta /V\right)\\ & -\frac{V}{\beta^{2}}\left[J(1-(\eta -iv)/V)-J(1-\eta /V)\right]\\ & ∼-\ln(1-iv/\eta )-J^{\prime }\left(1\right)\frac{iv}{\beta^{2}}+O\left(1/V\right)\\ & ∼-\ln(1-iv/\eta )+i\rho_{c}v+O\left(1/V\right), \end{array}$$

because, from Equations (54) and (58), it follows that $J^{\prime }\left(1\right)/\beta^{2}=-\rho_{c}$. Using now

(65) $$\xi^{-N^{\prime }}=e^{-N^{\prime }\ln\left(1+iv/V\right)}∼e^{-i\rho^{\prime }v},$$

where $\rho^{\prime }=N^{\prime }/V$, we have

(66) $$\begin{array}{cc} K(N^{\prime }|z) & ∼\frac{1}{2\pi V}\int_{-\infty }^{\infty }dv\;\frac{e^{-i(\rho^{\prime }-\rho_{c})v}}{1-iv/\eta }\\ & ∼-\frac{\eta }{2\pi iV}\int_{-\infty }^{\infty }dv\;\frac{e^{-i(\rho^{\prime }-\rho_{c})v}}{v+i\eta }\\ & ∼\Delta \rho^{\prime }\;\eta \;e^{-(\rho^{\prime }-\rho_{c})\;\eta }\;\theta \left(\rho^{\prime }-\rho_{c}\right), \end{array}$$

with $\Delta \rho^{\prime }=1/V$. This expression can be written in terms of ρ=N/V, expressing $\eta =V(1-ze^{-\beta \epsilon_{0}})$ and using Equation (60). Indeed, substituting s=1−η/V into Equation (60), we obtain

(67) $$\rho =\frac{1-\eta /V}{\eta }+\rho_{1}\left(1-\eta /V,\beta \right)∼\eta^{-1}+\rho_{c}\left(\beta \right)+O\left(1/V\right),$$

and replacing $\eta^{-1}=\rho -\rho_{c}$ in Equation (66), we finally have the explicit form of the kernel:

(68) $$K\left(\rho^{\prime }|\rho \right)\equiv K\left(N^{\prime }|z\right)/\Delta \rho^{\prime }=\frac{e^{-\left(\rho^{\prime }-\rho_{c}\right)/\left(\rho -\rho_{c}\right)}}{\rho -\rho_{c}}\;\theta \left(\rho^{\prime }-\rho_{c}\right),$$

valid for $\beta >\beta_{c}$. This result was obtained by Ziff et al. for the free ideal Bose gas in [17], and the kernel $K(\rho^{\prime }|\rho )$ is known as Kac density. In Appendix C, we give an alternative derivation of Equations (66) and (68) that makes use of the Laplace method.

The explicit expression of the kernel can be used to connect averages in the CE and GCE expressed in terms of ρ

(69) $${\langle f\rangle }_{GC}\left(\rho \right)=\int_{0}^{\infty }d\rho^{\prime }\;K\left(\rho^{\prime }|\rho \right)\;{\langle f\rangle }_{C}\left(\rho^{\prime }\right).$$

In particular, it is easy to show that $\int d\rho^{\prime }\;K\left(\rho^{\prime }|\rho \right)=1$, and that the following relation holds:

(70) $${\left(\rho -\rho_{c}\right)}^{k}=\int_{0}^{\infty }d\rho^{\prime }\;K\left(\rho^{\prime }|\rho \right)\frac{{\left(\rho^{\prime }-\rho_{c}\right)}^{k}}{k!}.$$

The above result will play an important role in the study of the occupation number fluctuations in the canonical and grand canonical ensembles.

Finally, we observe that, when expressed in the variable *z*, the kernel takes the form

(71) $$K\left(N^{\prime }|z\right)/\Delta \rho^{\prime }=V\left(1-s\right)\;e^{-V\left(1-s\right)\left(\rho^{\prime }-\rho_{c}\right)},$$

and therefore, in the limit V≫1 with 1−s=O(1), i.e., for η≫1, it reduces to

(72) $$K\left(N^{\prime }|z\right)=\Delta \rho^{\prime }\delta \left(\rho^{\prime }-\rho_{c}\right)=\delta^{Kr}\left(N^{\prime }-N_{c}\right),$$

where $\delta^{Kr}$ is the Kronecker delta. Thus, as expected, in the limit $\beta \to \beta_{c}^{-}$, we recover the expression (24) valid for $\beta <\beta_{c}$, i.e., in the non-condensed phase.

### 3.4. Density Fluctuations and Grand Canonical Catastrophe

As mentioned before, the explicit form of the Kac kernel allows us to connect averages in the GCE with those in the CE. Since the analytical expression of the Kac kernel is the same as in the case of the uniform system, the relations between density fluctuations in the CE and in the GCE coincide with those derived in [17]. We report here the formulae to keep the paper self-contained. In particular, the average number of bosons in the condensed phase in the GCE is given by Equation (48) and therefore one has, for large *V*,

(73) $${\langle {\hat{N}}_{0}\rangle }_{GC}=\frac{1}{s^{-1}-1}∼V\left(\rho -\rho_{c}\right),$$

where we have used Equation (67), and the dependence on *T* is through $\rho_{c}$. From the Relations (69) and (70), we then obtain that the averages of ${\hat{N}}_{0}$ are equal in the two ensembles

(74) $${\langle {\hat{N}}_{0}\rangle }_{C}={\langle {\hat{N}}_{0}\rangle }_{GC}.$$

On the contrary, considering the mean square occupation number, in the GCE, one has

(75) $${\langle {\hat{N}}_{0}^{2}\rangle }_{GC}={\langle {\hat{N}}_{0}\rangle }_{GC}+2{\langle {\hat{N}}_{0}\rangle }_{GC}^{2}=V\left(\rho -\rho_{c}\right)+2V^{2}{\left(\rho -\rho_{c}\right)}^{2}$$

and therefore

(76) $${\langle {\hat{N}}_{0}^{2}\rangle }_{GC}-{\langle {\hat{N}}_{0}\rangle }_{GC}^{2}=V\left(\rho -\rho_{c}\right)+V^{2}{\left(\rho -\rho_{c}\right)}^{2}.$$

Exploiting again the Relation (70), in the CE, one obtains

(77) $${\langle {\hat{N}}_{0}^{2}\rangle }_{C}={\langle {\hat{N}}_{0}\rangle }_{C}+{\langle {\hat{N}}_{0}\rangle }_{C}^{2}=V\left(\rho -\rho_{c}\right)+V^{2}{\left(\rho -\rho_{c}\right)}^{2},$$

and

(78) $${\langle {\hat{N}}_{0}^{2}\rangle }_{C}-{\langle {\hat{N}}_{0}\rangle }_{C}^{2}=V\left(\rho -\rho_{c}\right).$$

In particular, for the second-order correlation function (at zero time delay; see also Section 3.4), we have

(79) $$g^{\left(2\right)}\left(0\right)\equiv \frac{\langle {\hat{N}}_{0}^{2}\rangle -\langle {\hat{N}}_{0}\rangle }{{\langle {\hat{N}}_{0}\rangle }^{2}}=\left\{\begin{array}{cc} 1 & \mathrm{in}\;\mathrm{the}\;\mathrm{CE}\\ 2 & \mathrm{in}\;\mathrm{the}\;\mathrm{GCE}, \end{array}\right.$$

in agreement with what was observed in experiments [28]. Indeed, in Ref. [28] (see Figure 2b), experimental results are reported showing that $g^{\left(2\right)}\left(0\right)=2$, even in the low-temperature phase, if a larger reservoir size is considered, which corresponds to the realization of a genuine grand canonical conditions. On the other hand, when canonical conditions are realized, the same figure shows that fluctuations are damped, and $g^{\left(2\right)}\left(0\right)=1$ is observed.

Moreover, for the intensive density fluctuations, one has

(80) $$\lim_{V\to \infty }\frac{{\langle {\hat{N}}_{0}^{2}\rangle }_{C}-{\langle {\hat{N}}_{0}\rangle }_{C}^{2}}{V^{2}}=0\;\;\mathrm{in}\;\mathrm{the}\;\mathrm{CE},$$

while

(81) $$\lim_{V\to \infty }\frac{{\langle {\hat{N}}_{0}^{2}\rangle }_{GC}-{\langle {\hat{N}}_{0}\rangle }_{GC}^{2}}{V^{2}}={\left(\rho -\rho_{c}\right)}^{2}\;\;\mathrm{in}\;\mathrm{the}\;\mathrm{GCE}.$$

The latter result is known as a grand canonical catastrophe [17] because of the counter-intuitive phenomenon of macroscopic fluctuations, non-vanishing in the low temperature limit T→0. This finding shows the inequivalence of ensembles at the level of fluctuations and has been interpreted as an example where the GCE is not appropriate for computing averages [17,18,19,20,21]. However, in the experimental setup with photons in the microcavity, where GCE conditions are realized, macroscopic fluctuations have been actually observed [14], making the prediction of the GCE physically consistent [24].

The mathematical origin of such huge fluctuations can be traced back to the specific form of the Kac kernel connecting the CE and GCE, as detailed in the previous subsection.

#### Spatial Density–Density Correlation Function of the Condensate

We now discuss another quantity that is affected by the grand canonical catastrophe: the spatial density–density correlation function of the Bose–Einstein condensate.

We start by noting that, for both the CE and GCE, the one-body correlation function $\langle {\hat{\psi }}^{+}\left(r\right)\hat{\psi }\left(r^{\prime }\right)\rangle$ can be written in terms of ladder operators in this way:

(82) $$\begin{array}{ccc} \langle {\hat{\psi }}^{+}\left(r\right)\hat{\psi }\left(r^{\prime }\right)\rangle & = & \sum_{m,m^{\prime }}\langle {\hat{a}}_{m}^{+}{\hat{a}}_{m^{\prime }}\rangle \phi_{m}^{*}\left(r\right)\phi_{m^{\prime }}\left(r^{\prime }\right)\\ & = & \langle {\hat{a}}_{0}^{+}{\hat{a}}_{0}\rangle \phi_{0}^{*}\left(r\right)\phi_{0}\left(r^{\prime }\right)+\ldots \\ & = & \langle {\hat{N}}_{0}\rangle \phi_{0}^{*}\left(r\right)\phi_{0}\left(r^{\prime }\right)+\ldots \;, \end{array}$$

where the dots represent the contributions of the excited states.

Working at zero temperature, where the effect due to the excited states goes to zero, we find that

(83) $$\langle {\hat{\psi }}^{+}\left(r\right)\hat{\psi }\left(r^{\prime }\right)\rangle =\langle {\hat{N}}_{0}\rangle \phi_{0}^{*}\left(r\right)\phi_{0}\left(r^{\prime }\right)\;.$$

Previously, we have seen that, at zero temperature,

(84) $$\langle {\hat{N}}_{0}\rangle =N$$

for both the CE and GCE. Thus,

(85) $$\langle {\hat{\psi }}^{+}\left(r\right)\hat{\psi }\left(r^{\prime }\right)\rangle =N\;\phi_{0}^{*}\left(r\right)\phi_{0}\left(r^{\prime }\right)\;.$$

Recalling Equation (30), namely $\phi_{0}\left(r\right)=e^{-{\left|r\right|}^{2}/\left(2l_{H}^{2}\right)}/\left(\pi^{1/2}l_{H}\right)$ with $l_{H}=\sqrt{\hbar /(m_{eff}\omega )}$, we eventually obtain

(86) $$\langle {\hat{\psi }}^{+}\left(r\right)\hat{\psi }\left(r^{\prime }\right)\rangle =\frac{N}{\pi l_{H}^{2}}\;e^{-{\left(|r\right|}^{2}+|r^{\prime }{|}^{2})/\left(2l_{H}^{2}\right)}\;.$$

Setting $r^{\prime }=0$, the previous expression becomes

(87) $$\langle {\hat{\psi }}^{+}\left(r\right)\hat{\psi }\left(0\right)\rangle =\frac{N}{\pi l_{H}^{2}}\;e^{-{\left|r\right|}^{2}/\left(2l_{H}^{2}\right)},$$

while, setting $r^{\prime }=r$, we obtain the result

(88) $$\langle {\hat{\psi }}^{+}\left(r\right)\hat{\psi }\left(r\right)\rangle =\langle \hat{\rho }\left(r\right)\rangle =\frac{N}{\pi l_{H}^{2}}\;e^{-{\left|r\right|}^{2}/l_{H}^{2}}\;.$$

We then study the density–density correlation function $\langle \hat{\rho }\left(r\right)\hat{\rho }\left(r^{\prime }\right)\rangle$, which, for both the CE and GCE, can be written in terms of ladder operators as follows:

(89) $$\begin{array}{ccc} \langle \hat{\rho }\left(r\right)\hat{\rho }\left(r^{\prime }\right)\rangle & = & \sum_{m,m^{\prime },m^{\prime \prime },m^{\prime \prime \prime }}\langle {\hat{a}}_{m}^{+}{\hat{a}}_{m^{\prime }}{\hat{a}}_{m^{\prime \prime }}^{+}{\hat{a}}_{m^{\prime \prime \prime }}\rangle \\ & \times & \phi_{m}^{*}\left(r\right)\phi_{m^{\prime }}\left(r\right)\phi_{m^{\prime \prime }}^{*}\left(r^{\prime }\right)\phi_{m^{\prime \prime \prime }}\left(r^{\prime }\right)\\ & = & \langle {\hat{a}}_{0}^{+}{\hat{a}}_{0}{\hat{a}}_{0}^{+}{\hat{a}}_{0}\rangle |\phi_{0}\left(r\right){|}^{2}{\left|\phi_{0}\left(r^{\prime }\right)\right|}^{2}+\ldots \\ & = & \langle {\hat{N}}_{0}^{2}\rangle |\phi_{0}\left(r\right){|}^{2}{\left|\phi_{0}\left(r^{\prime }\right)\right|}^{2}+\ldots \;, \end{array}$$

where the dots stand for the contributions of the excited states. Similarly, we have

(90) $$\begin{array}{ccc} \langle \hat{\rho }\left(r\right)\rangle \langle \hat{\rho }\left(r^{\prime }\right)\rangle & = & \sum_{m,m^{\prime },m^{\prime \prime },m^{\prime \prime \prime }}\langle {\hat{a}}_{m}^{+}{\hat{a}}_{m^{\prime }}\rangle \langle {\hat{a}}_{m^{\prime \prime }}^{+}{\hat{a}}_{m^{\prime \prime \prime }}\rangle \\ & \times & \phi_{m}^{*}\left(r\right)\phi_{m^{\prime }}\left(r\right)\phi_{m^{\prime \prime }}^{*}\left(r^{\prime }\right)\phi_{m^{\prime \prime \prime }}\left(r^{\prime }\right)\\ & = & \langle {\hat{a}}_{0}^{+}{\hat{a}}_{0}\rangle \langle {\hat{a}}_{0}^{+}{\hat{a}}_{0}\rangle |\phi_{0}\left(r\right){|}^{2}{\left|\phi_{0}\left(r^{\prime }\right)\right|}^{2}+\ldots \\ & = & {\langle {\hat{N}}_{0}\rangle }^{2}|\phi_{0}\left(r\right){|}^{2}{\left|\phi_{0}\left(r^{\prime }\right)\right|}^{2}+\ldots \;. \end{array}$$

Thus, considering the zero temperature limit for simplicity and neglecting the effect due to the excited states, we obtain

(91) $$\begin{array}{ccc} \langle \hat{\rho }\left(r\right)\hat{\rho }\left(r^{\prime }\right)\rangle -\langle \hat{\rho }\left(r\right)\rangle \langle \hat{\rho }\left(r^{\prime }\right)\rangle & = & \left(\langle {\hat{N}}_{0}^{2}\rangle -{\langle {\hat{N}}_{0}\rangle }^{2}\right)\\ & \times & |\phi_{0}{\left(r\right)|}^{2}{\left|\phi_{0}\left(r^{\prime }\right)\right|}^{2}\;. \end{array}$$

By using our previous results of Equations (80) and (81), which crucially depend on the statistical ensemble, at zero temperature, we then have

(92) $$\frac{\langle {\hat{N}}_{0}^{2}\rangle -{\langle {\hat{N}}_{0}\rangle }^{2}}{V^{2}}=\left\{\begin{array}{cc} 0 & \mathrm{in}\;\mathrm{the}\;\mathrm{CE}\\ \rho^{2} & \mathrm{in}\;\mathrm{the}\;\mathrm{GCE}. \end{array}\right.$$

As a consequence, for the density–density correlation function *per particle*, we finally obtain

(93) $$\frac{\langle \hat{\rho }\left(r\right)\hat{\rho }\left(r^{\prime }\right)\rangle -\langle \hat{\rho }\left(r\right)\rangle \langle \hat{\rho }\left(r^{\prime }\right)\rangle }{N}=0$$

in the CE, and

(94) $$\frac{\langle \hat{\rho }\left(r\right)\hat{\rho }\left(r^{\prime }\right)\rangle -\langle \hat{\rho }\left(r\right)\rangle \langle \hat{\rho }\left(r^{\prime }\right)\rangle }{N}=\frac{N}{\pi^{2}l_{H}^{4}}e^{-{\left(|r\right|}^{2}+|r^{\prime }{|}^{2})/l_{H}^{2}},$$

in the GCE. Equation (94) is not translationally invariant, as expected, due to the presence of the confining harmonic potential. Moreover, setting $r^{\prime }=0$, we obtain

(95) $$\frac{\langle \hat{\rho }\left(r\right)\hat{\rho }\left(0\right)\rangle -\langle \hat{\rho }\left(r\right)\rangle \langle \hat{\rho }\left(0\right)\rangle }{N}=\left\{\begin{array}{cc} 0 & \mathrm{in}\;\mathrm{the}\;\mathrm{CE}\\ \frac{N}{\pi^{2}l_{H}^{4}}e^{-{\left|r\right|}^{2}/l_{H}^{2}} & \mathrm{in}\;\mathrm{the}\;\mathrm{GCE}, \end{array}\right.$$

showing a decay to zero for a large |r| with a correlation length $l_{H}$, representing the size of the bosonic cloud, that diverges in the thermodynamic limit as ω→0. Note that the prefactor in the above expression remains finite in the thermodynamic limit.

### 3.5. Internal Energy and Specific Heat

To complete the description of the thermodynamic properties of the system, we compute the internal energy and the specific heat in the normal and condensed phases. These quantities have been measured in experiments in [12], where a comparison with a numerical evaluation of the Bose–Einstein distribution function is reported. Here, we provide close analytical expressions for average energy and specific heat, valid both above and below the critical temperature (see also ref. [29] for a similar computation in a 3D system).

#### 3.5.1. Grand Canonical Ensemble

The (average) internal energy of the system in the GCE is given by

(96) $$\begin{array}{cc} E_{\mathrm{GC}}\left(\beta ,z\right) & =-\frac{\partial }{\partial \beta }{|}_{z}\ln Z_{\mathrm{GC}}\left(\beta ,z\right)\\ & =\frac{\partial }{\partial s}{|}_{\beta }\left[\ln\left(1-s\right)+\frac{1}{{\left(\beta \hbar \omega \right)}^{2}}J\left(s\right)\right]\;\frac{\partial }{\partial \beta }{|}_{z}s\\ & +\frac{\partial }{\partial \beta }{|}_{s}\frac{1}{{\left(\beta \hbar \omega \right)}^{2}}J\left(s\right)\\ & =\bar{N}\left(\beta ,z\right)\;\epsilon_{0}-2\frac{\beta^{-3}}{{\left(\hbar \omega \right)}^{2}}\;J\left(s\right), \end{array}$$

where we have used

(97) $$\frac{\partial }{\partial \beta }{|}_{z}s=-\epsilon_{0}\;s,$$

and

(98) $$\begin{array}{ccc} & - & s\;\frac{\partial }{\partial s}{|}_{\beta }\left[\ln\left(1-s\right)+\frac{1}{{\left(\beta \hbar \omega \right)}^{2}}J\left(s\right)\right]=\\ & = & z\;\frac{\partial }{\partial z}{|}_{\beta }\ln Z_{\mathrm{GC}}\left(\beta ,z\right)=\bar{N}\left(\beta ,z\right)={\langle \hat{N}\rangle }_{\mathrm{GC}}. \end{array}$$

Note that zero-point energy goes to zero in the thermodynamic limit. Here, we keep this term because we take into account first-order corrections.

Evaluating the derivative, we have the explicit form

(99) $$\bar{N}\left(\beta ,z\right)=\frac{s}{1-s}+\frac{1}{{\left(\beta \hbar \omega \right)}^{2}}I\left(s\right).$$

In the above expressions, J(s) and I(s) are defined in Equations (51) and (54), respectively. Now using the condition ${\langle \hat{N}\rangle }_{\mathrm{GC}}=N$, we have

(100) $$E_{\mathrm{GC}}\left(\beta ,z\right)=N\;\epsilon_{0}-2\frac{\beta^{-3}}{{\left(\hbar \omega \right)}^{2}}\;J\left(s\right).$$

When $\beta >\beta_{c}$, i.e., $T<T_{c}$, the variable *s* saturates to s=1, so we have

(101) EGC(β,N)=Nℏω+2ζ(3)(ℏω)2β−3,β>βcxxNℏω−2J(s)(ℏω)2β−3,β<βc

where we have used J(1)=−ζ(3). In the regime $\beta <\beta_{c}$, the variable s≡s(β,N) is obtained from

(102) $$\frac{1}{{\left(\beta \hbar \omega \right)}^{2}}I\left(s\right)=N,\;\Rightarrow \;I\left(s\right)=N{\left(\beta \hbar \omega \right)}^{2}.$$

Using

(103) $$\frac{1}{\hbar \omega }=\sqrt{\frac{N}{I(1)}}\;\beta_{c}=\sqrt{\frac{6N}{\pi^{2}}}\;\beta_{c},$$

we can express (101) in terms of *N* and $T_{c}$. A simple calculation leads to

(104) EGCNTc=π6[1N1/2+126π3ζ(3)T/Tc3],T<Tcxxπ6[1N1/2−126π3J(s)T/Tc3],T>Tc

where $s=s(T,T_{c})$ for $T>T_{c}$ is the solution of $I\left(s\right)=\left(\pi^{2}/6\right){\left(T_{c}/T\right)}^{2}$.

Having the expression of the (average) internal energy, we can compute the specific heat. From (101), we have, for $\beta >\beta_{c}$,

(105) $$\frac{\partial }{\partial \beta }{|}_{N}E_{\mathrm{GC}}\left(\beta ,N\right)=-6\frac{\zeta (3)}{{\left(\hbar \omega \right)}^{2}}\;\beta^{-4}$$

which, using $\left(\partial /\partial T\right)=-\beta^{2}\left(\partial /\partial \beta \right)$, leads to

(106) $$\frac{\partial }{\partial T}{|}_{N}E_{\mathrm{GC}}\left(T,N\right)=6\frac{\zeta (3)}{{\left(\hbar \omega \right)}^{2}}\;T^{2}=6N\frac{\zeta (3)}{I(1)}\;{\left(\frac{T}{T_{{\;\;}_{c}}}\right)}^{2},$$

Then,

(107) $$c\left(T\right)=\frac{1}{N}\frac{\partial }{\partial T}{|}_{N}E_{\mathrm{GC}}\left(T,N\right)=6\frac{\zeta (3)}{I(1)}\;{\left(\frac{T}{T_{{\;\;}_{c}}}\right)}^{2}$$

or

(108) $$c\left(T\right)=\frac{36\;\zeta (3)}{\pi^{2}}\;{\left(\frac{T}{T_{{\;\;}_{c}}}\right)}^{2},\;T<T_{c}.$$

This analytical result is in agreement with the very recent finding of ref. [30].

For temperature $T>T_{c}$, the calculation is a bit more involved. Again from (101), we have, for $\beta <\beta_{c}$,

(109) $$\begin{array}{cc} \frac{\partial }{\partial \beta }{|}_{N}E_{\mathrm{GC}}\left(\beta ,N\right) & =6\frac{J(s)}{{\left(\hbar \omega \right)}^{2}}\;\beta^{-4}-2\frac{J^{\prime }\left(s\right)}{{\left(\hbar \omega \right)}^{2}}\;\beta^{-3}\;\frac{\partial }{\partial \beta }{|}_{N}s\\ & =6\frac{J(s)}{{\left(\hbar \omega \right)}^{2}}\;\beta^{-4}+2\frac{I(s)}{s{\left(\hbar \omega \right)}^{2}}\;\beta^{-3}\;\frac{\partial }{\partial \beta }{|}_{N}s \end{array}$$

or, using (102),

(110) $$\frac{\partial }{\partial \beta }{|}_{N}E_{\mathrm{GC}}\left(\beta ,N\right)=6\frac{J(s)}{{\left(\hbar \omega \right)}^{2}}\;\beta^{-4}+2\frac{N}{s}\;\beta^{-1}\;\frac{\partial }{\partial \beta }{|}_{N}s.$$

Now from (102), for N=const, we have

(111) $$-2\frac{I(s)}{{\left(\hbar \omega \right)}^{2}}\beta^{-3}\;d\beta +\frac{I^{\prime }\left(s\right)}{{\left(\beta \hbar \omega \right)}^{2}}\;ds=0,$$

and then

(112) $$\frac{\partial }{\partial \beta }{|}_{N}s=2\frac{I(s)}{I^{\prime }\left(s\right)}\beta^{-1},$$

with

(113) $$\begin{array}{c} I^{\prime }\left(s\right)=\frac{d}{ds}\int_{0^{+}}^{+\infty }dy\;\frac{y}{s^{-1}e^{y}-1}=\int_{0^{+}}^{+\infty }dy\;\frac{y\;e^{-y}}{{\left(1-s\;e^{-y}\right)}^{2}}\\ =-\frac{1}{s}\ln\left(1-s\right). \end{array}$$

Then,

(114) $$\frac{\partial }{\partial \beta }{|}_{N}E_{\mathrm{GC}}\left(\beta ,N\right)=6\frac{J(s)}{{\left(\hbar \omega \right)}^{2}}\;\beta^{-4}-4N\frac{I(s)}{\ln(1-s)}\;\beta^{-2},$$

which, with (102), gives

(115) $$\frac{\partial }{\partial T}{|}_{N}E_{\mathrm{GC}}\left(T,N\right)=-6\frac{J(s)}{{\left(\hbar \omega \right)}^{2}}\;\beta^{-2}+4N^{2}\frac{{\left(\beta \hbar \omega \right)}^{2}}{\ln(1-s)},\;T>T_{c}.$$

so that, for $T>T_{c}$,

(116) $$c\left(T\right)=\frac{1}{N}\frac{\partial }{\partial T}{|}_{N}E_{\mathrm{GC}}\left(T,N\right)=-6\frac{J(s)}{N{\left(\beta \hbar \omega \right)}^{2}}+4N\frac{{\left(\beta \hbar \omega \right)}^{2}}{\ln(1-s)}.$$

If we finally express ℏω in terms of *N* and $T_{c}$, i.e., using Equation (103), we obtain

(117) $$c\left(T\right)=-6\frac{J(s)}{I(1)}{\left(\frac{T}{T_{c}}\right)}^{2}+4\frac{I(1)}{\ln(1-s)}{\left(\frac{T_{c}}{T}\right)}^{2},\;T>T_{c},$$

where s=s(T,N) is again the solution of (102). Note that our Equation (117) also appears in ref. [30].

**Figure 1:**
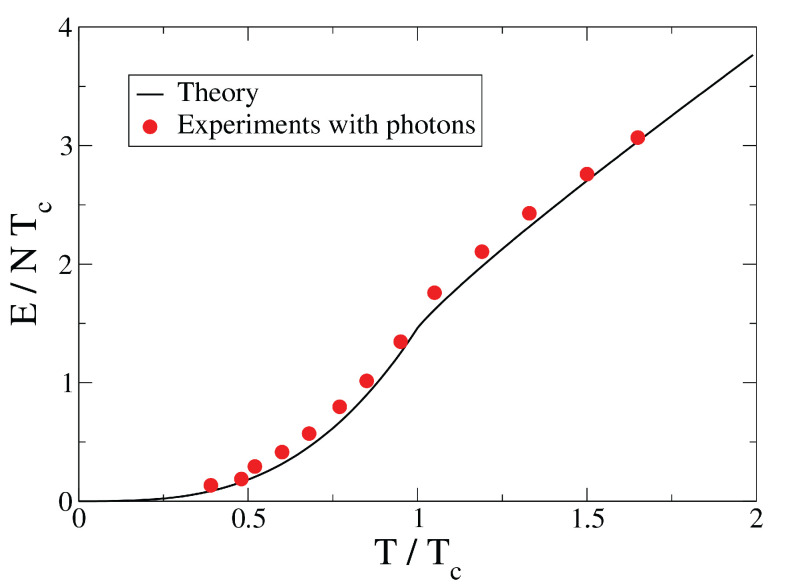
Internal energy *E* as a function of temperature *T*. Here, $T_{c}$ is the critical temperature of Bose–Einstein condensation. Solid line: our analytical theory in the grand canonical ensemble, Equation (104) in the limit N→∞. Filled circles: experimental data of ref. [12].

**Figure 2:**
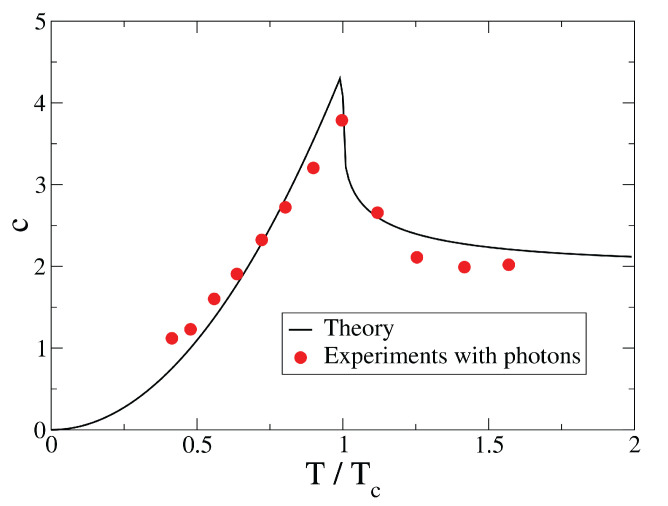
Specific heat *c* as a function of temperature. $T_{c}$ is the critical temperature. Solid line: our analytical results, Equations (108) and (117) with N→∞, in the grand canonical ensemble. Filled circles: experimental data of ref. [12].

In Figure 1 and Figure 2, we plot the rescaled average energy $E/(NT_{c})$ and the specific heat *c* as a function of $T/T_{c}$, respectively, and compare the analytical predictions with experimental data taken from ref. [12]. There is a remarkable good agreement between our analytical results and the experimental ones. Notice that in Ref. [12] are also reported theoretical curves, obtained with a numerical procedure, that are very similar to the solid curves of our Figure 1 and Figure 2. The two figures strongly suggest that these experiments with photons are in a regime where the thermodynamic limit is practically achieved.

#### 3.5.2. Canonical Ensemble

The same expression of the average internal energy, and therefore of the specific heat, also hold in the CE. Let us prove it.

From Equation (9), we know that

(118) $$\begin{array}{ccc} {\langle f\rangle }_{\mathrm{GC}}\left(s\right) & = & \sum_{N^{\prime }\geq 0}{\langle f\rangle }_{C}\left(N^{\prime }\right)\;K\left(N^{\prime }|s\right)\\ & = & \sum_{N^{\prime }\geq 0}\Delta \rho^{\prime }\;{\langle f\rangle }_{C}\left(N^{\prime }\right)\;\frac{K(N^{\prime }|s)}{\Delta \rho^{\prime }}, \end{array}$$

where $\Delta \rho^{\prime }=1/V$; thus, in the thermodynamic limit, we have

(119) $${\langle f\rangle }_{\mathrm{GC}}\left(\rho \right)=\int_{0}^{+\infty }d\rho^{\prime }\;{\langle f\rangle }_{C}\left(\rho^{\prime }\right)\;K\left(\rho^{\prime }|\rho \right),$$

where s=s(ρ)=1−η/V with $\eta^{-1}=\rho -\rho_{c}$, and

(120) $$K\left(\rho^{\prime }|\rho \right)\equiv K\left(N^{\prime }|s\right)/\Delta \rho^{\prime }=\frac{e^{-\left(\rho^{\prime }-\rho_{c}\right)/\left(\rho -\rho_{c}\right)}}{\rho -\rho_{c}}\;\theta \left(\rho^{\prime }-\rho_{c}\right).$$

From Equation (104), neglecting the first term irrelevant for N→∞, we have

(121) $$\frac{E}{NT_{c}}=-\frac{2}{I(1)}{\left(\frac{T}{T_{c}}\right)}^{3}\;J\left(s(\rho )\right).$$

Then, substituting

(122) $$\begin{array}{cc} J(1-\eta /V) & ∼J\left(1\right)-J^{\prime }\left(1\right)\;\eta /V+O\left(1/V^{2}\right)\\ & ∼J\left(1\right)+I\left(1\right)\;\eta /V.+O\left(1/V^{2}\right),\;V\to \infty , \end{array}$$

it follows that, in the grand canonical ensemble,

(123) $$\frac{E_{\mathrm{GC}}}{NT_{c}}∼\frac{12}{\pi^{2}}\zeta \left(3\right){\left(\frac{T}{T_{c}}\right)}^{3}-2{\left(\frac{T}{T_{c}}\right)}^{3}\;\frac{1}{V(\rho -\rho_{c})}+O\left(1/V^{2}\right),$$

where we have used J(1)=−ζ(3) and $I\left(1\right)=\pi^{2}/6$.

Using this expression in Equation (119) with the explicit form of the kernel, we obtain

(124) $$\begin{array}{c} \int_{\rho_{c}}^{+\infty }d\rho^{\prime }\frac{e^{-\left(\rho^{\prime }-\rho_{c}\right)/\left(\rho -\rho_{c}\right)}}{\rho -\rho_{c}}\frac{E_{C}\left(\rho^{\prime }\right)}{NT_{c}}\\ ∼\frac{E_{0}}{NT_{c}}-2{\left(\frac{T}{T_{c}}\right)}^{3}\;\frac{1}{V(\rho -\rho_{c})}+O\left(1/V^{2}\right) \end{array}$$

where

(125) $$\frac{E_{0}}{NT_{c}}=\frac{12}{\pi^{2}}\zeta \left(3\right){\left(\frac{T}{T_{c}}\right)}^{3}.$$

Now, using the normalization of $K(\rho^{\prime }|\rho )$,

(126) $$\int_{\rho_{c}}^{+\infty }d\rho^{\prime }\frac{e^{-\left(\rho^{\prime }-\rho_{c}\right)/\left(\rho -\rho_{c}\right)}}{\rho -\rho_{c}}=1,$$

it follows that

(127) $$\frac{E_{C}\left(\rho^{\prime }\right)}{NT_{c}}∼\frac{E_{0}}{NT_{c}}-2{\left(\frac{T}{T_{c}}\right)}^{3}\;\frac{1}{V}\;\delta \left(\rho^{\prime }-\rho_{c}\right)+O\left(1/V^{2}\right),$$

as one can check by substituting back the above expression into Equation (124). Recalling that $1/V=\Delta \rho^{\prime }$ and using the identity

(128) $$\Delta \rho^{\prime }\delta \left(\rho^{\prime }-\rho_{c}\right)=\delta^{\mathrm{Kr}}\left(N^{\prime }-N_{c}\right),$$

we have

(129) $$\frac{E_{C}\left(N^{\prime }\right)}{NT_{c}}∼\frac{E_{0}}{NT_{c}}-2{\left(\frac{T}{T_{c}}\right)}^{3}\;V\;\delta^{\mathrm{Kr}}\left(N^{\prime }-N_{c}\right)+O\left(1/V^{2}\right).$$

The number *N* in the grand canonical ensemble and $N^{\prime }$ in the canonical ensemble are related by the requirement $N\equiv {\langle \hat{N}\rangle }_{\mathrm{GC}}=N^{\prime }$; hence, the Kronecker delta vanishes and we conclude that

(130) $$\frac{E_{C}\left(N,T\right)}{NT_{c}}=\frac{E_{\mathrm{GC}}\left(N,T\right)}{NT_{c}}=\frac{12}{\pi^{2}}\zeta \left(3\right){\left(\frac{T}{T_{c}}\right)}^{3},\;T<T_{c}.$$

in the thermodynamic limit.

## 4. Conclusions

We have discussed a general formalism to derive physical quantities in the canonical ensemble from the corresponding ones in the grand canonical ensemble, where the calculations are usually much simpler. Then, motivated by recent experiments with photons, we have applied this formalism to the study of an ideal Bose gas of particles under harmonic confinement in two spatial dimensions. Quite remarkably, also working in the thermodynamic limit, density fluctuations and spatial density–density correlations of the Bose–Einstein condensate display a strongly different behavior in the two ensembles. Similar to previous predictions for the uniform Bose gas [17], for the non-uniform condensate, we find that the density–density correlation is zero in the canonical ensemble and non-zero in the grand canonical ensemble. This result, which is known as the grand canonical catastrophe because of the counter-intuitive phenomenon of non-vanishing macroscopic fluctuations in the low temperature limit T→0, turns out to be a real phenomenon as it has in fact been observed in experiments with photons in the microcavity [14]. Our study sheds new light on the underlying mathematical and physical mechanisms that induce this intriguing behavior. In the last part of this paper, we have also obtained analytical formulas for the internal energy and the specific heat both in the condensed phase and in the normal phase. Similar results, obtained with a fully numerical procedure, can be found in ref. [12]. For these quantities, we have provided explicit analytical expressions, including also finite-size effects. The comparison with the experimental data of ref. [12] shows a good agreement between our analytical theory and the empirical results.

## Data Availability

The data presented in this study are available on request from the corresponding author.

## Appendix A

By substituting the expression (8), we obtain

$${\langle f\rangle }_{\mathrm{GC}}\left(z\right)=\sum_{N}{\langle f\rangle }_{C}\left(N\right)\;\frac{z^{N}\;Z_{C}\left(N\right)}{Z_{\mathrm{GC}}\left(z\right)},$$

and hence

$${\langle f\rangle }_{\mathrm{GC}}\left(z\right)\;Z_{\mathrm{GC}}\left(z\right)=\sum_{N}z^{N}\;Z_{C}\left(N\right)\;{\langle f\rangle }_{C}\left(N\right).$$

Inverting this relation gives

$${\langle f\rangle }_{C}\left(N\right)\;Z_{C}\left(N\right)=\frac{1}{2\pi i}\;\oint_{\Gamma }dz\;z^{-1-N}{\langle f\rangle }_{\mathrm{GC}}\left(z\right)\;Z_{\mathrm{GC}}\left(z\right)$$

and hence

(A1) $${\langle f\rangle }_{C}\left(N\right)=\frac{1}{2\pi i}\;\oint_{\Gamma }\frac{dz}{z}{\langle f\rangle }_{\mathrm{GC}}\left(z\right)\;\frac{z^{-N}Z_{\mathrm{GC}}\left(z\right)}{Z_{C}\left(N\right)}.$$

Recalling the expression of K(N|z), we finally obtain the following relation

(A2) $${\langle f\left(\sigma \right)\rangle }_{C}\left(N\right)=\frac{1}{2\pi i}\;\oint_{\Gamma }\frac{dz}{z}\frac{{\langle f\left(\sigma \right)\rangle }_{\mathrm{GC}}\left(z\right)}{K(N|z)}$$

between averages in the GCE and averages in the CE.

From the expression (8), which involves both $Z_{C}\left(N\right)$ and $Z_{\mathrm{GC}}\left(z\right)$, it seems that, in order to evaluate K(N|z), the partition functions in both ensembles should be explicitly known. However, an alternative approach would be to introduce the generating function

(A3) $$\begin{array}{ccc} \hat{K}\left(\xi |z\right) & = & \sum_{N}\xi^{N}\;K\left(N|z\right)=\frac{1}{Z_{\mathrm{GC}}\left(z\right)}\sum_{N}\xi^{N}\;z^{N}\;Z_{C}\left(N\right)\\ & = & \frac{Z_{\mathrm{GC}}\left(\xi z\right)}{Z_{\mathrm{GC}}\left(z\right)}, \end{array}$$

so that

(A4) $$\begin{array}{ccc} K(N|z) & = & \frac{1}{2\pi i}\oint_{\Gamma }d\xi \;\xi^{-1-N}\hat{K}\left(\xi |z\right)\\ & = & \frac{1}{2\pi i}\oint_{\Gamma }d\xi \;\xi^{-1-N}\frac{Z_{\mathrm{GC}}\left(\xi z\right)}{Z_{\mathrm{GC}}\left(z\right)} \end{array}$$

is expressed in terms of $Z_{\mathrm{GC}}\left(z\right)$ only. This expression can also be obtained by substituting the expression (5) into the definition (8):

(A5) $$K\left(N|z\right)=\frac{z^{N}}{Z_{\mathrm{GC}}\left(z\right)}\frac{1}{2\pi i}\oint_{\Gamma }ds\;s^{-1-N}\;Z_{\mathrm{GC}}\left(s\right),$$

and setting s=ξz

(A6) $$K\left(N|z\right)=\frac{1}{Z_{\mathrm{GC}}\left(z\right)}\frac{1}{2\pi i}\oint_{\Gamma }d\xi \;\xi^{-1-N}\;Z_{\mathrm{GC}}\left(\xi z\right).$$

Finally, let us mention that the same result can also be obtained by introducing the discrete Fourier transform

(A7) $$\bar{K}\left(\xi |z\right)=\sum_{N}e^{iN\xi /V}\;K\left(N|z\right)=\frac{Z_{\mathrm{GC}}\left(ze^{i\xi /V}\right)}{Z_{\mathrm{GC}}\left(z\right)},$$

or, in terms of the chemical potential μ,

(A8) $$\bar{K}\left(\xi |\mu \right)=\frac{Z_{\mathrm{GC}}\left(\mu +iT\xi /V\right)}{Z_{\mathrm{GC}}\left(z\right)}.$$

In this case, it should be noted that

(A9) $$K\left(N|z\right)=\int_{-\infty }^{+\infty }\frac{d\xi }{2\pi }\;e^{-i\xi x}\;\bar{K}\left(\xi |z\right),$$

with x=N/V, is not the inverse of (A7) because

(A10) $$\int_{-\infty }^{+\infty }\frac{d\xi }{2\pi }\;e^{-i\xi x}=\delta \left(x\right),$$

where δ(x) is the Dirac delta function. If, however, the sum in (A7) is dominated by N≫1, as in the thermodynamic limit, then x=N/V can be considered a “continuous” variable and the sum can be replaced by an integral so that (A9) becomes meaningful.

## Appendix B

Consider

(A11) $$Z_{C}\left(N\right)=\frac{1}{2\pi i}\oint_{\Gamma }dzz^{-1-N}\;Z_{\mathrm{GC}}\left(z\right)=\frac{1}{2\pi i}\oint_{\Gamma }\frac{dz}{z}\;e^{N\psi (z)},$$

with

(A12) $$\psi \left(z\right)=-\ln z+\frac{1}{N}\ln Z_{\mathrm{GC}}\left(z\right).$$

In the limit N≫1, the integral is dominated by the neighborhood of the point $z^{*}$:

(A13) $$\psi^{\prime }\left(z^{*}\right)=-\frac{1}{z^{*}}+\frac{1}{N}\frac{\partial }{\partial z^{*}}\ln Z_{\mathrm{GC}}\left(z^{*}\right)=0,$$

which leads to the saddle point equation:

(A14) $$z^{*}\frac{\partial }{\partial z^{*}}\ln Z_{\mathrm{GC}}\left(z^{*}\right)=N.$$

Since $Z_{C}\left(N\right)$ is real, the saddle point lies on the real axis, $ℑz^{*}=0$. A simple calculation then shows that

(A15) $$\begin{array}{ccc} \psi^{\prime \prime }\left(z^{*}\right) & = & \frac{1}{{z^{*}}^{2}}+\frac{1}{N}\;\frac{\partial^{2}}{\partial z^{2}}\;\ln Z_{\mathrm{GC}}\left(z\right){|}_{z=z^{*}}\\ & = & \frac{1}{{z^{*}}^{2}N}\;z^{*}\frac{\partial }{\partial z^{*}}\left[z^{*}\frac{\partial }{\partial z^{*}}\ln Z_{\mathrm{GC}}\left(z^{*}\right)\right]\\ & = & \frac{1}{{z^{*}}^{2}N}\;\left[{\langle N^{2}\rangle }_{\mathrm{GC}}\left(z^{*}\right)-{\langle N\rangle }_{\mathrm{GC}}{\left(z^{*}\right)}^{2}\right]>0. \end{array}$$

Then, by expanding ψ(z) about $z^{*}$ up to second order, as in Equation (18), and integrating along the steepest descent path parallel to the imaginary axis $z=z^{*}+iy$, −∞<y<+∞, we obtain

(A16) $$Z_{C}\left(N\right)∼\sqrt{\frac{\sigma_{\psi }^{2}}{2\pi }}\frac{e^{N\psi (z^{*})}}{z^{*}}=\sqrt{\frac{\sigma_{\psi }^{2}}{2\pi }}\;{z^{*}}^{-1-N}\;Z_{\mathrm{GC}}\left(z^{*}\right),N\gg 1,$$

where $z^{*}=z\left(N\right)$ is the solution of (A14) and we have defined $\sigma_{\psi }^{-2}=N\psi^{\prime \prime }\left(z^{*}\right)>0$. Dropping sub-leading terms as N≫1, the expression (A16) implies that

(A17) $$\ln Z_{C}\left(N\right)=\ln Z_{\mathrm{GC}}\left(z\right)-N\ln z,\;N\gg 1.$$

with $z=z^{*}=z\left(N\right)$ the solution of (A14).

Equations (A14) and (A17) imply that, in the thermodynamic limit N≫1, $\ln Z_{C}\left(N\right)$ is the Legendre transform of $\ln Z_{\mathrm{GC}}\left(z\right)$ with respect to lnz, and hence it is the inverse Legendre transform of (21) and (22). Note also that (A14) implies that, in the thermodynamic limit,

(A18) $${\langle \hat{N}\rangle }_{\mathrm{GC}}\left(z^{*}\right)=N={\langle \hat{N}\rangle }_{C}.$$

Finally, for the kernel $K{\left(N|z\right)}^{-1}$, we have

(A19) $$K{\left(N|z\right)}^{-1}=\frac{z^{-N}\;Z_{\mathrm{GC}}\left(z\right)}{Z_{C}\left(N\right)}=e^{N\psi (z)}{\left[\frac{1}{2\pi i}\oint_{\Gamma }\frac{dz}{z}e^{N\psi (z)}\right]}^{-1}.$$

In the thermodynamic limit, using (A16), we have

(A20) $$K{\left(N|z\right)}^{-1}∼\sqrt{\frac{2\pi }{\sigma_{\psi }^{2}}}\;z^{*}\;e^{\frac{1}{2\sigma_{\psi }^{2}}{\left(z-z^{*}\right)}^{2}}=\sqrt{\frac{2\pi }{\sigma_{\psi }^{2}}}\;z^{*}\;e^{-\frac{1}{2\sigma_{\psi }^{2}}\;y^{2}},$$

with $y=-i(z-z^{*})$. This expression can be simplified further by recalling that $\sigma_{\psi }^{2}=O\left(1/N\right)$. Thus,

(A21) $$K{\left(N|z\right)}^{-1}∼\frac{2\pi z^{*}}{\sqrt{2\pi \sigma_{\psi }^{2}}}\;e^{-\frac{1}{2\sigma_{\psi }^{2}}\;y^{2}}∼2\pi z^{*}\;\delta \left(y\right),\;N\gg 1,$$

and, using δ(ax)=(1/a)δ(x), we finally have

(A22) $$K{\left(N|z\right)}^{-1}∼2\pi i\;z\;\delta \left(z-z(N)\right),\;N\gg 1.$$

with z(N) the solution of (A17). Note that

(A23) $$\frac{1}{2\pi i}\oint_{\Gamma }\frac{dz}{z}\;K{\left(N,z\right)}^{-1}=1,$$

as follows from the definition of K(N|z).

## Appendix C

In the derivation of the Kac kernel, the assumption s=1−η/V may appear ad hoc. Therefore, here, we present an alternative derivation of (66). The starting point is Equation (61), i.e.,

(A24) $$K\left(N|z\right)=\frac{z^{N}}{Z_{\mathrm{GC}}\left(\beta ,V,z\right)}\frac{1}{2\pi i}\oint_{\Gamma }d\xi \;\xi^{-1-N}\;Z_{\mathrm{GC}}\left(\beta ,V,\xi \right),$$

where, here, we use *N* instead of $N^{\prime }$. The integral in this equation is the canonical partition function (see (5)),

(A25) $$Z_{C}\left(\beta ,V,N\right)=\frac{1}{2\pi i}\oint_{\Gamma }d\xi \;\xi^{-1-N}\;Z_{\mathrm{GC}}\left(\beta ,V,\xi \right),$$

where Γ is a closed curve in the complex ξ-plane encircling the origin and not crossing the real axis on the cut $ℜ\;\xi >e^{\beta \epsilon_{0}}$.

If $\epsilon_{0}\neq 0$, it is useful to define $s=e^{-\beta \epsilon_{0}}z=\alpha_{0}z$ so that

(A26) $$Z_{C}\left(\beta ,V,N\right)=\frac{\alpha_{0}^{N}}{2\pi i}\oint_{\Gamma }ds\;s^{-1-N}\;Z_{\mathrm{GC}}\left(\beta ,V,\alpha_{0}^{-1}s\right),$$

where, now, Γ is a closed curve in the complex *s*-plane encircling the origin and not crossing the real axis on the cut ℜs>1.

In our specific case, we have (see (50))

(A27) $$Z_{\mathrm{GC}}\left(\beta ,V,\alpha_{0}^{-1}s\right)=-\frac{1}{1-s}\;e^{-\frac{V}{\beta^{2}}J\left(s\right)},$$

where J(s) is given in (51). Inserting this expression into (A26), we have

(A28) $$Z_{C}\left(\beta ,V,N\right)=\frac{\alpha_{0}^{N}}{2\pi i}\oint_{\Gamma }\frac{ds}{s}\;\frac{e^{V\phi (s)}}{1-s},$$

where

(A29) $$\phi \left(s\right)=-\rho \;\ln s-\frac{1}{\beta^{2}}J\left(s\right),$$

where ρ=N/V is the density in the canonical ensemble.

In the thermodynamic limit V→∞, N→∞ with ρ=constant, the integral is dominated by the saddle point $s^{*}$. Assuming that $1-s^{*}=O\left(1\right)$ as V→∞, the saddle point is given by $\phi^{\prime }\left(s^{*}\right)=0$, where

(A30) $$\phi^{\prime }\left(s\right)=-\frac{1}{s}\left[\rho -\rho_{1}\left(\beta ,s\right)\right],$$

where, from (53),

(A31) $$\rho_{1}\left(\beta ,s\right)=-\frac{s}{\beta^{2}}\;J^{\prime }\left(s\right)=\frac{1}{\beta^{2}}\;I\left(s\right),$$

with I(s) given in (54). The saddle point is thus the solution of

(A32) $$\rho_{1}\left(\beta ,s^{*}\right)=\rho .$$

Since I(1)<∞, it follows that

(A33) $$\rho_{1}\left(\beta ,s\right)\leq \rho_{1}\left(\beta ,1\right)\equiv \rho_{c}\left(\beta \right)=\frac{1}{\beta^{2}}\;I\left(1\right).$$

As a consequence, the saddle point Equation (A32) does not have a solution for fixed ρ if $\beta >\beta_{c}\left(\rho \right)$ with

(A34) $$\beta_{c}\left(\rho \right):\;\rho_{c}\left(\beta_{c}\right)=\rho ,$$

or, alternatively, for fixed β if $\rho >\rho_{c}\left(\beta \right)$.

To study this regime, we consider

(A35) $$\begin{array}{cc} \bar{\phi }\left(s\right) & =-\rho \;\ln s+\frac{1}{V}\ln\;Z_{\mathrm{GC}}\left(\beta ,\alpha_{0}^{-1}s\right)\\ & =-\frac{1}{V}\ln\left(1-s\right)+\phi \left(s\right). \end{array}$$

A simple calculation shows that

(A36) $${\bar{\phi }}^{\prime }\left(s\right)=-\frac{1}{s}\left[\rho -\bar{\rho }\left(\beta ,s\right)\right],$$

where

(A37) $$\bar{\rho }\left(\beta ,s\right)=\frac{1}{V}\bar{N}\left(\beta ,V,s\right)=\frac{s}{V}\frac{\partial }{\partial s}\ln\;Z_{\mathrm{GC}}\left(\beta ,\alpha_{0}^{-1}s\right).$$

As a consequence, in the thermodynamic limit, the leading contribution to the integral

(A38) $$Z_{C}\left(\beta ,V,N\right)=\frac{\alpha_{0}^{N}}{2\pi i}\oint_{\Gamma }\frac{ds}{s}\;e^{V\bar{\phi }\left(s\right)}$$

comes from the region close to the saddle point $s^{*}$ given by

(A39) $$\bar{\rho }\left(\beta ,s^{*}\right)=\rho .$$

Substituting (A35), the saddle point equation becomes

(A40) $${\bar{\phi }}^{\prime }\left(\beta ,s^{*}\right)=\frac{1}{V}\frac{1}{1-s^{*}}+\phi^{\prime }\left(s^{*}\right)=0$$

which, using (A30), gives

(A41) $$\frac{1}{V}\frac{s^{*}}{1-s^{*}}=\rho -\rho_{1}\left(\beta ,s^{*}\right).$$

From this, we see that, if $\rho >\rho_{c}\left(\beta \right)$, then $1-s^{*}$ must be positive and, moreover, $1-s^{*}=O\left(1/V\right)$ as V→∞. From (A41), it follows that

(A42) $$s^{*}=1-\frac{1}{V}\frac{s^{*}}{\rho -\rho_{1}\left(\beta ,s^{*}\right)},$$

which, for V→∞, gives

(A43) $$s^{*}∼1-\frac{1}{V}\frac{1}{\rho -\rho_{c}\left(\beta \right)}+O\left(1/V^{2}\right),\;V\to \infty .$$

Since $s^{*}<1$, in (A28), we can integrate over the circle $C^{*}:s=s^{*}e^{i\theta }$; thus, in the limit V→∞,

(A44) $$\begin{array}{cc} Z_{C}\left(\beta ,V,N\right)=\frac{\alpha_{0}^{N}}{2\pi i}\oint_{C^{*}}\frac{ds}{s}\;\frac{e^{V\phi (s)}}{1-s} & ∼\frac{\alpha_{0}^{N}}{2\pi i}\int_{s=s^{*}e^{i\theta },\left|\theta \right|<\epsilon }\frac{ds}{s}\;\frac{e^{V\phi (s)}}{1-s}\\ & ∼\frac{\alpha_{0}^{N}}{2\pi i}\int_{s=s^{*}+iy,\left|y\right|<\epsilon }\frac{ds}{s}\;\frac{e^{V\phi (s)}}{1-s}, \end{array}$$

where ϵ is a small (arbitrary) parameter used to isolate the relevant region of integration. Since ϵ is small, we can expand ϕ(s) as

(A45) $$\phi \left(s^{*}+iy\right)∼\phi \left(s^{*}\right)+\phi^{\prime }\left(s^{*}\right)iy-\frac{1}{2}\phi^{\prime \prime }\left(s^{*}\right)y^{2}+O\left(y^{3}\right),$$

where, from (A40) and (A43),

(A46) $$\phi^{\prime }\left(s^{*}\right)=-\frac{1}{\eta },\;\eta^{-1}=\rho -\rho_{c}\left(\beta \right),$$

while

(A47) $$\phi^{\prime \prime }\left(s^{*}\right)=-\frac{1}{s^{*}}\phi^{\prime }\left(s^{*}\right)+\frac{1}{s}\frac{\partial }{\partial s}\rho_{1}\left(\beta ,s\right){|}_{s=s^{*}}>0.$$

Substituting, we then have, as V→∞,

(A48) $$Z_{C}\left(\beta ,V,N\right)∼\frac{\alpha_{0}^{N}}{2\pi }\int_{-\infty }^{+\infty }\frac{dy}{1-\eta /V+iy}\;\frac{e^{V\left[\phi \left(s^{*}\right)-iy/\eta -\frac{1}{2}\phi^{\prime \prime }y^{2}\right]}}{\eta /V-iy}\left[1+O(Vy^{3})\right].$$

The proposed approach follows that described by Dingle in Ref. [31], where he performs an additional change in variable, namely, takes $y=t/\sqrt{V\phi^{\prime \prime }}-i\eta /V$ and expands the term $O(Vy^{3})$ and ${\left(1-\eta /V+iy\right)}^{-1}$ in powers of *t*. This is equivalent to taking $s=1+it/\sqrt{V\phi^{\prime \prime }}$ as the integration path.Since we are only interested in the leading term of $\ln Z_{C}\left(\beta ,V,N\right)/V$ as V→∞, this step is not necessary, and we can just expand in powers of $V^{-1}$. Thus, we have

(A49) $$Z_{C}\left(\beta ,V,N\right)∼\frac{\alpha_{0}^{N}e^{V\phi (s^{*})}}{-2\pi i}\int_{-\infty }^{+\infty }dy\;y^{-1}\;e^{-iyV/\eta -\frac{V}{2}\phi^{\prime \prime }y^{2}}+\ldots ,$$

where the dots denote sub-leading terms as V→∞. By rescaling $y\to y/\sqrt{V\phi^{\prime \prime }}$, we finally arrive at

(A50) $$Z_{C}\left(\beta ,V,N\right)∼\frac{\alpha_{0}^{N}e^{V\phi (s^{*})}}{-2\pi i}\int_{-\infty }^{+\infty }dy\;y^{-1}\;e^{iya-\frac{1}{2}y^{2}}+\ldots ,$$

where $a=-\sqrt{V/\phi^{\prime \prime }}/\eta$. The integral can be expressed in terms of the the parabolic cylinder function $D_{-1}\left(a\right)$:

(A51) $$\int_{\infty }^{+\infty }\frac{dy}{y}e^{-\frac{1}{2}y^{2}+iay}=-i\sqrt{2\pi }\;e^{a^{2}/4}D_{-1}\left(a\right),$$

so that

(A52) $$Z_{C}\left(\beta ,V,N\right)∼\frac{\alpha_{0}^{N}e^{V\phi (s^{*})}}{\sqrt{2\pi }}\;e^{a^{2}/4}D_{-1}\left(a\right)+\ldots .$$

In the limit V→∞, the term *a* is large and negative, $D_{-1}\left(a\right)∼e^{-a^{2}/4}$, and hence

(A53) $$Z_{C}\left(\beta ,V,N\right)∼\frac{\alpha_{0}^{N}e^{V\phi (s^{*})}}{\sqrt{2\pi }}+\ldots ,\;V\to \infty .$$

Finally,

(A54) $$\begin{array}{cc} \phi (s^{*}) & =-\rho \ln s^{*}-\frac{1}{\beta^{2}}J\left(s^{*}\right)\\ & ∼-\rho \ln\left(1-\eta /V\right)-\frac{1}{\beta^{2}}J\left(1-\eta /V\right)\\ & ∼\rho \eta /V-\frac{1}{\beta^{2}}J\left(1\right)+\frac{1}{\beta^{2}}J^{\prime }\left(1\right)\eta /V+\ldots \end{array}$$

But, $J^{\prime }\left(1\right)/\beta^{2}=-\rho_{c}\left(\beta \right)$; thus,

(A55) $$\begin{array}{cc} \phi (s^{*}) & ∼\left[\rho -\rho_{c}\left(\beta \right)\right]\eta /V-\frac{1}{\beta^{2}}J\left(1\right)+\ldots \\ & ∼1/V-\frac{1}{\beta^{2}}J\left(1\right)+\ldots . \end{array}$$

In conclusion, the leading term $\ln Z_{C}\left(\beta ,V,N\right)/V$ as V→∞ is

(A56) $$Z_{C}\left(\beta ,V,N\right)∼\alpha_{0}^{N}\;e^{-\frac{V}{\beta^{2}}\;J\left(1\right)}\;V\to \infty .$$

We can now go back to (A24), which, using (A56), becomes

(A57) $$K\left(N|z\right)∼\frac{{\left(z\alpha_{0}\right)}^{N}}{Z_{\mathrm{GC}}\left(\beta ,V,z\right)}\;e^{-\frac{V}{\beta^{2}}\;J\left(1\right)},\;V\to \infty .$$

Now, recalling that the fugacity *z* is related to ${\langle N\rangle }_{\mathrm{GC}}$ via (62),

(A58) $${\left(z\alpha_{0}\right)}^{N}=e^{N\ln(z\alpha_{0})}∼e^{N\ln(1-\bar{\eta }/V)}∼e^{-\rho \;\bar{\eta }},$$

because $\alpha_{0}z=1-\bar{\eta }/V$ with $\eta^{-1}=\bar{\rho }-\rho_{c}\left(\beta \right)$. Similarly,

(A59) $$\begin{array}{cc} e^{-\frac{V}{\beta^{2}}\;J\left(1\right)}Z_{\mathrm{GC}}{\left(\beta ,V,s\right)}^{-1} & =\left(1-\alpha_{0}z\right)e^{-\frac{V}{\beta^{2}}\left[J\left(1\right)-J\left(z\alpha_{0}\right)\right]}\\ & ∼\frac{\bar{\eta }}{V}\;e^{-\frac{V}{\beta^{2}}J^{\prime }\left(1\right)\;\bar{\eta }/V}∼\frac{\bar{\eta }}{V}\;e^{-\rho_{c}\left(\beta \right)\;\bar{\eta }},\;V\to \infty . \end{array}$$

Thus, collecting all the terms we have, cfr. (66) and (68),

(A60) $$\begin{array}{cc} K\left(N|z\right)=K\left(\rho |\bar{\rho }\right)\;\Delta \rho & ∼\frac{\bar{\eta }}{V}\;e^{-\left[\rho -\rho_{c}\right]\;\bar{\eta }}\\ & ∼\Delta \rho \;\frac{e^{-\left(\rho -\rho_{c}\right)/\left(\bar{\rho }-\rho_{c}\right)}}{\bar{\rho }-\rho_{c}},\;\rho >\rho_{c}\left(\beta \right),\;V\to \infty . \end{array}$$

where Δρ=1/V.

## References

[1] Anderson M., Ensher J., Matthews M., Wieman C., Cornell E. (1995). Observation of Bose-Einstein condensation in a dilute atomic vapor. Science.

[2] Davis K., Mewes M.O., Andrews M., van Druten N., Durfee D., Kurn D., Ketterle W. (1995). Bose-Einstein condensation in a gas of sodium atoms. Phys. Rev. Lett..

[3] Bradley C., Sackett C., Tollett J., Hulet R. (1995). Evidence of Bose-Einstein condensation in an atomic gas with attractive interactions. Phys. Rev. Lett..

[4] Cornell E.A., Wieman C.E. (2002). Nobel Lecture: Bose-Einstein condensation in a dilute gas, the first 70 years and some recent experiments. Rev. Mod. Phys..

[5] Leggett A.J. (2001). Bose-Einstein condensation in the alkali gases: Some fundamental concepts. Rev. Mod. Phys..

[6] Dalfovo F., Giorgini S., Pitaevskii L.P., Stringari S. (1999). Theory of Bose-Einstein condensation in trapped gases. Rev. Mod. Phys..

[7] Fetter A.L. (2009). Rotating trapped bose-einstein condensates. Rev. Mod. Phys..

[8] Zapf V., Jaime M., Batista C. (2014). Bose-Einstein condensation in quantum magnets. Rev. Mod. Phys..

[9] Klaers J., Schmitt J., Vewinger F., Weitz M. (2010). Bose–Einstein condensation of photons in an optical microcavity. Nature.

[10] Klaers J., Schmitt J., Damm T., Vewinger F., Weitz M. (2011). Bose–Einstein condensation of paraxial light. Appl. Phys. B.

[11] Schmitt J., Damm T., Dung D., Vewinger F., Klaers J., Weitz M. (2014). Observation of grand-canonical number statistics in a photon Bose-Einstein condensate. Phys. Rev. Lett..

[12] Damm T., Schmitt J., Liang Q., Dung D., Vewinger F., Weitz M., Klaers J. (2016). Calorimetry of a Bose–Einstein-condensed photon gas. Nat. Commun..

[13] Damm T., Dung D., Vewinger F., Weitz M., Schmitt J. (2017). First-order spatial coherence measurements in a thermalized two-dimensional photonic quantum gas. Nat. Commun..

[14] Schmitt J. (2018). Dynamics and correlations of a Bose–Einstein condensate of photons. J. Phys. B At. Mol. Opt. Phys..

[15] Öztürk F.E., Vewinger F., Weitz M., Schmitt J. (2023). Fluctuation-dissipation relation for a Bose-Einstein condensate of photons. Phys. Rev. Lett..

[16] Campa A., Dauxois T., Ruffo S. (2009). Statistical mechanics and dynamics of solvable models with long-range interactions. Phys. Rep..

[17] Ziff R.M., Uhlenbeck G.E., Kac M. (1977). The ideal Bose-Einstein gas, revisited. Phys. Rep..

[18] Holthaus M., Kalinowski E., Kirsten K. (1998). Condensate fluctuations in trapped Bose gases: Canonical vs. microcanonical ensemble. Ann. Phys..

[19] Fujiwara I., Ter Haar D., Wergeland H. (1970). Fluctuations in the population of the ground state of Bose systems. J. Stat. Phys..

[20] Kocharovsky V.V., Kocharovsky V.V., Holthaus M., Ooi C.R., Svidzinsky A., Ketterle W., Scully M.O. (2006). Fluctuations in ideal and interacting Bose–Einstein condensates: From the laser phase transition analogy to squeezed states and Bogoliubov quasiparticles. Adv. At. Mol. Opt. Phys..

[21] Yukalov V. (2007). Bose-Einstein condensation and gauge symmetry breaking. Laser Phys. Lett..

[22] Crisanti A., Sarracino A., Zannetti M. (2019). Condensation versus ordering: From the spherical models to Bose-Einstein condensation in the canonical and grand canonical ensemble. Phys. Rev. Res..

[23] Berlin T.H., Kac M. (1952). The spherical model of a ferromagnet. Phys. Rev..

[24] Zannetti M. (2015). The grand canonical catastrophe as an instance of condensation of fluctuations. Europhys. Lett..

[25] Holmes M.H. (2012). Introduction to Perturbation Methods.

[26] Kac M., Thompson C.J. (1977). Correlation functions in the spherical and mean spherical models. J. Math. Phys..

[27] Salasnich L. (2017). Quantum Physics of Light and Matter.

[28] Klaers J., Schmitt J., Damm T., Vewinger F., Weitz M. (2012). Statistical physics of Bose-Einstein-condensed light in a dye microcavity. Phys. Rev. Lett..

[29] Grossmann S., Holthaus M. (1995). *λ*-transition to the Bose-Einstein condensate. Zeit. Naturforschung A.

[30] Morales-Amador M.I., Romero-Rochin V., Paredes R. (2024). Critical exponents and fluctuations at BEC in a 2D harmonically trapped ideal gas. J. Phys. B At. Mol. Opt. Phys..

[31] Dingle R.B. (1973). Asymptotic Expansions.

